# Compression Test of Soft Food Gels Using a Soft Machine with an Artificial Tongue

**DOI:** 10.3390/foods8060182

**Published:** 2019-05-29

**Authors:** Kaoru Kohyama, Sayaka Ishihara, Makoto Nakauma, Takahiro Funami

**Affiliations:** 1 Food Research Institute, National Agriculture and Food Research Organization (NARO), 2-1-12 Kannondai, Tsukuba, Ibaraki 305-8642, Japan; 2 San-Ei Gen F. F. I., Inc., 1-1-11 Sanwa-Cho, Toyonaka, Osaka 561-8588, Japan; sayaka-ishihara@saneigenffi.co.jp (S.I.); m-nakauma@saneigenffi.co.jp (M.N.); tfunami@saneigenffi.co.jp (T.F.)

**Keywords:** texture, compression test, artificial tongue, fracture, soft machine, gellan gum gels, care food

## Abstract

Care food is increasingly required in the advanced-aged society. Mechanical properties of such foods must be modified such that the foods are easily broken by the tongue without chewing. When foods are compressed between the tongue and the hard palate, the tongue deforms considerably, and only soft foods are broken. To simulate tongue compression of soft foods, artificial tongues with stiffness similar to that of the human tongue were created using clear soft materials. Model soft gels were prepared using gellan gums. A piece of gel on an artificial tongue was compressed using a texture analyzer. The deformation profile during the compression test was obtained using a video capture system. The soft machine equipped a soft artificial tongue sometimes fractured food gels unlike hard machine, which always fracture gels. The fracture properties measured using the soft machine were better than those obtained from a conventional test between hard plates to mimic natural oral processing in humans. The fracture force on foods measured using this soft machine may prove useful for the evaluation of food texture that can be mashed using the tongue.

## 1. Introduction

Aging has progressed worldwide. The requirement of appropriate care foods for the elderly in our aged society has also increased. Care foods for individuals who have difficulty in mastication must be soft enough to be consumed by compression with the tongue and hard palate without chewing. Japan is the leading country in the context of aging populations, as the number of elderly people aged 65 years and over in Japan is about 34 million, and the percentage of elderly individuals in the population was 27.3% in 2016 [1]. The elderly population will further increase and reach approximately 40 million by 2042. In contrast, the total population has decreased since 2007. Since 2012, the Japanese Ministry of Agriculture, Forestry, and Fisheries has promoted new care foods for people with dysphagia, difficulty in mastication, malnutrition, and future frailty [2]. Smile Care Foods with a red/yellow/blue mark are being promoted to aid consumers in choosing suitable food products in each area of a storefront [2]. One of the categories included in Smile Care Foods (yellow 3) is the tongue-mashable level [2]. However, to date, numerical values for tongue-mashable foods have not been presented. The aim of this study is to determine tongue mashability of soft food gels using a new instrumental test.

The tongue pressure of humans has been widely measured [3,4,5,6,7,8,9,10,11,12,13,14,15,16]. As balloon-type sensors are handy and easy to manage, significant data for various ages, gender, and eating abilities have been accumulated and correlated with other activities [10,11,12]. The maximum voluntary tongue pressure of healthy adults was reported as 40–100 kPa in these reports, which decreases with age [13]. Subjects with tongue pressure lower than 40 kPa represent reduced tongue strength [14], thus some people may require special care foods [2].

When a soft food is compressed between the tongue and hard palate, the tongue deforms considerably to fracture the food. The tongue tenses during food compression and may become 5–10 times harder than in the relaxed state [16,17]. Elastic modulus is a mechanical parameter as the ratio of force per unit area required for small deformation expressed as strain (ratio of deformation to the initial length) of samples, which indicates stiffness or difficulty in deformation. In our previous study [16], apparent elastic moduli of the human tongue of healthy young subjects were measured. The value was determined at approximately 20% compressive strain. The apparent modulus was 12.2 ± 4.2 kPa and 122.5 ± 58.5 kPa in a relaxed and tense state, respectively. To simulate tongue compression, multiple artificial tongues with apparent modulus similar to that of the human tongue were used to compress soft gels between one of the artificial tongues and metal plate [16,18]. Agar gels with fracture strain of ca. 60% but different fracture forces were broken when the deformation of agar gel was greater than that of the artificial tongue [16]. This suggests that decreased stiffness expressed as decreased modulus or increased deformability of the artificial tongue did not facilitate fracture the food gels. Among several materials tested, an artificial tongue with the apparent modulus of 55 kPa was most suitable to simulate the human behavior. Specifically, softer food gels with fracture stress (force per unit area) less than ca. 50 kPa or a lower fracture force of cylindrical gels (20 mm diameter) of 20 N could be consumed easily using the tongue and palate [16]. When gels with similar fracture forces and different fracture strains were compared, highly deformable gels with greater fracture strain were not broken by the artificial tongue [18]. Although gels having a fracture strain of 70% or over had lower elastic modulus and fracture stress [19], fracture strain became more critical to change the oral processing method from tongue–palate compression to chewing [18].

The artificial tongues were made using silicone rubber in our previous studies [16,18]. The artificial tongue had to be of a size similar to that of the food gel (20 mm in diameter and 10 mm high), and the compression test was stopped at 10 mm after the surface of the gel contacted the upper plate. When gels deformed more than the artificial tongue in the vertical direction, the gels were fractured between the artificial tongue and metal plate [16,18]. However, highly deformable gels were not broken under these conditions [18]. The wider artificial tongue (56 mm in diameter) was suggested to be more suitable to simulate tongue compression of gels with a high fracture strain; however, observation at the fracture point was not possible. The limited test conditions were caused because the silicone rubber was not transparent. The real human tongue is wider than the food gel and may compress the gel more than the gel’s initial height (10 mm) during tongue deformation. Observations of deformation of food gels and artificial tongues were difficult as the tongue covered the food gel during compression. Thus, new artificial tongues were prepared with transparent soft materials, and soft gels as model foods as in previous studies [18,19,20,21] were compressed between the soft material and hard plate. We tested three urethane gels with varying stiffness (slightly higher, comparable, and lower level) of the human tongue. We report here a pilot study on gel fracture using a soft machine with transparent artificial tongues.

## 2. Materials and Methods

Model food gels (φ20 × 10 mm) were prepared by mixing low- and high-acylated gellan gums (KELCOGEL^TM^ and KELCOGEL^TM^ LT-100, respectively, San-Ei Gen F. F. I., Inc., Osaka, Japan) as previously described [18,19,20,21] with slight modification. The concentrations of gellan gums are shown in Table 1. Sucrose (10% *w*/*w*), a food color (SAN GREEN^TM^ GC-EM, San-Ei Gen F. F. I., 0.2% *w*/*w*) and calcium lactate (0.1% *w*/*w*) were contained in all gels. The food gels were stored in a refrigerator and used within a month after preparation.

Artificial tongues were made using urethane transparent gels (HITOHADA^TM^ gel clear type, Exseal Co., Ltd., Mino, Gifu, Japan). Rectangular gels (50 × 50 × 10 mm) and cylindrical gels (φ13 × 10 mm) were prepared, and Asker C hardness was determined as 0, 7, and 15 by the manufacturer. They were named as H0, H7, and H15, hereafter.

Hardness of artificial tongues with 50 × 50 × 10 mm was determined with a durometer (Asker FP type, Kobunshi Keiki Co., Ltd., Kyoto, Japan) at 20 °C. Compression test of the cylindrical urethane gels was conducted using a TA.XT*plus* Texture Analyser with a 50 N load cell (Stable Micro Systems, Surrey, UK). A cylindrical specimen was compressed between a flat alminum plate and stage at a constant rate of 10 mm/s and 20 °C [18,19,20,21]. Both the plate and stage were made of aluminum. Compression started from 10 mm above, force was first detected when the upper plate contacted the surface of artificial tongue and increased during compression. The compression stopped at 2.5 mm from the contact. Apparent modulus was determined based on the stress value at 20% compression [18]. Force value at 2 mm deformation (F_2_) was read using an Exponent software (ver. 6.1.15.0, Stable Micro Systems). Apparent modulus in kPa was calculated as F_2_ (N)/initial sectional area (133 mm^2^)/2 (mm) × initial height (measured for each specimen, ca. 10 mm) × 1000.

**Figure 1:**
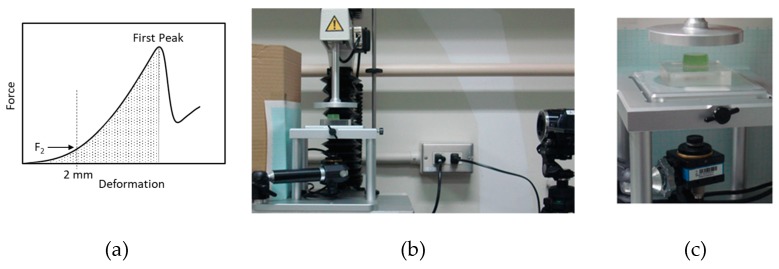
Typical force–deformation curve of a gellan gel (**a**) and setting for compression test of soft foods between an artificial tongue and plate (**b**,**c**). (**b**) Whole setup and (**c**) enlarged view around the sample. A food gel (φ20 × 10 mm) on a transparent artificial tongue (50 × 50 × 10 mm) was placed on a glass plate of a TA.XT*plus* Texture Analyser. Upper plate connected to a load cell was moved downward at a constant speed, and sample deformation was observed from the bottom and/or from the right side by video cameras during the compression test.

Fracture properties of food gels were measured under similar conditions using the Texture Analyser. Fracture force and deformation were determined from the first peak of the compression curve as shown in Figure 1a. Fracture strain was obtained as the ratio of the fracture deformation to the initial height of gel.

A soft machine was constructed using one of the rectangular artificial tongues and the texture analyzer as shown in Figure 1b. The bottom plate of the Texture Analyser was replaced by a glass plate. A food gel was placed on a rectangle artificial tongue set on the glass plate (Figure 1c) and compressed under similar conditions as above. The F_2_, fracture force and fracture deformation were obtained as above. The apparent modulus was calculated assuming the sectional area as 314 mm^2^, and fracture work was estimated as shaded area (N.mm) in Figure 1a.

The deformation profile during the compression test was obtained using a Video Capture Synchronisation System (Stable Micro Systems) as in the previous study [19]. Ratios of the cross-sectional area of food gels at fracture point to the initial area were determined by snapshots taken at closest to the fracture point and immediately prior to compression from the bottom video. True fracture stress was calculated as the fracture force divided by the cross-sectional area at the fracture point assuming the initial area of the gels as 314 mm^2^ [19]. The compression was stopped when the upper plate (P/75, Stable Micro Systems) contacted the upper surface of the artificial tongue and the load value reached 45 N.

In some cases, deformation of the food gel and artificial tongue was captured from the side using a video camera (Handycam, HDR-XR550V, Sony, Tokyo, Japan) as in the previous reports [16,18] (Figure 1b).

Statistical analyses were performed using a software package (SPSS ver. 23, IBM, Armonk, NY, USA). Statistical significance was set at *p* < 0.05. Mean values for different samples were tested using a one-way analysis of variance (ANOVA) followed by Tukey’s multiple comparisons as a *post-hoc* test, as appropriate.

## 3. Results

### 3.1. Gellan Gum Gels

Fracture force and strain of food gels measured between metal plates were shown in Table 2. These six gels were prepared to have a low (about 16 N) or high (about 24 N) fracture force, and a low (about 49%), middle (63%) or high (75%), respectively, fracture strain. The nomination is in accordance with a previous study [16].

### 3.2. Artificial Tongues

**Figure 2:**
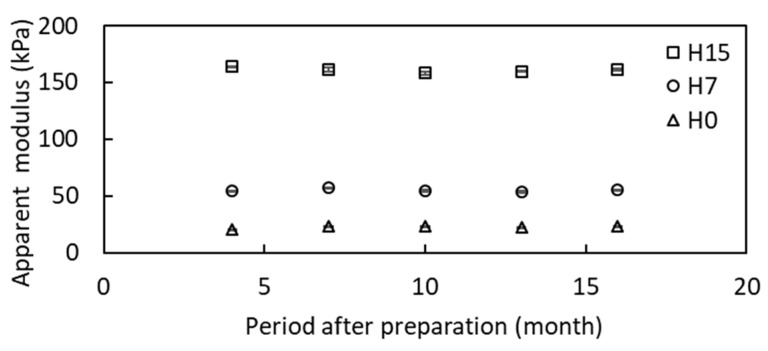
Apparent modulus of urethane gel for artificial tongue. Error bars represent standard errors of 2–7 samples.

The mechanical properties of the artificial tongues are shown in Table 3. As these gels did not fracture, the apparent modulus values determined at 20% compression were given. The artificial gels were stable after the compression test and recovered, even after food gels or the of texture analyzer probe (φ20) were inserted. The apparent modulus at 20 °C was measured repeatedly to test long-time stability at room temperature. As shown in Figure 2, they were similar for over a year, and variance values were small. According to the manufacturer, heat tolerance of the urethane gels is between −30 and 80 °C. Therefore, the test could be conducted at body temperature.

### 3.3. Compression of Gellan Gum Gels Placed on An Artificial Tongue

Gellan gels on an artificial tongue were compressed using a soft machine as shown in Figure 1. When softest artificial tongue H0 was used, harder gellan gels (A20, BC20, and D20) were not broken, while softer gels with 16 N fracture force sometimes fractured (Table 4). Fracture probability decreased as the fracture strain increased (A15 > BC15 > D15). H7 and H15 fractured all food gels as shown in Table 4.

**Figure 3:**
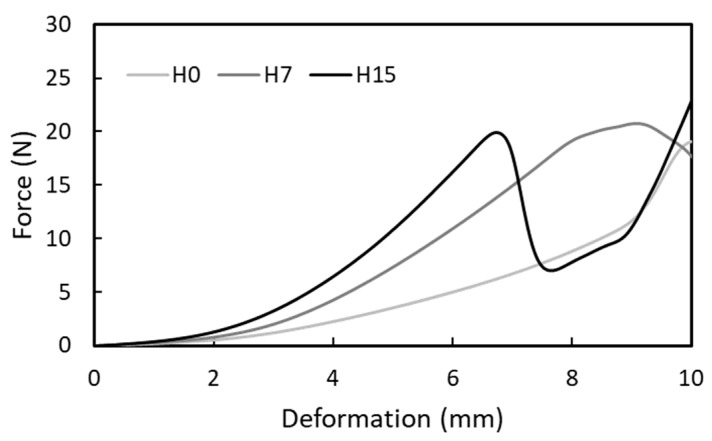
Examples of compression curves of food gel (BC20) on artificial tongues.

Figure 3 shows compression curves of a food gel (BC20) on different artificial tongues. The force values at a given deformation before fracture decreased as stiffness of the artificial tongues decreased. A soft machine equipped with an artificial tongue H15 showed a sharp peak at fracture as observed in the compression test using a hard machine. The fracture point became less sharp around 9 mm for the middle artificial tongue H7. The food gel never fractured using the softest H0 as shown in Table 4.

**Figure 4:**
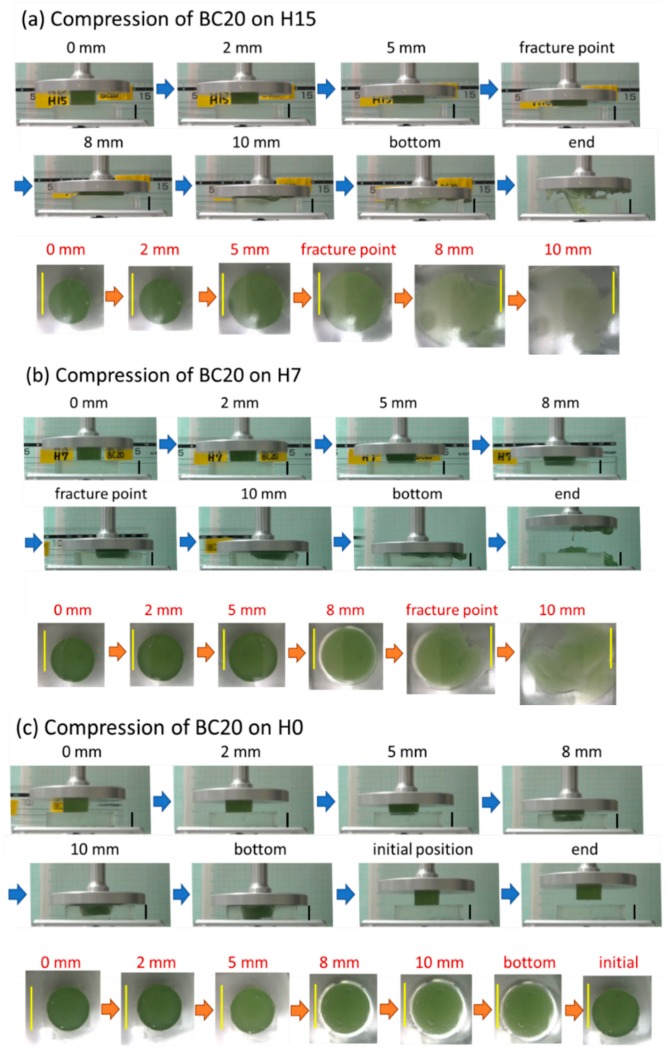
Examples of a food gel on soft material (i.e. as artificial tongue) observed from the side and bottom. Compression process of a BC gel specimen on (**a**) H15, (**b**) H7, and (**c**) H0 artificial tongue. Compression distance from the first contact to the food gel is shown over each snapshot. “Bottom” represents the moment that compression was stopped due to the limit force, “initial position” is the moment that the probe returned to 0 mm height, and “end” indicates the test completion. Black bars are 10 mm as original height of food and artificial tongue gels in the side views, and yellow bars are 20 mm as original diameter of food gels in the bottom views.

When food gels were compressed with the soft machine, phenomena different from those for the conventional test using a hard machine were observed. The transparent material was useful for direct observation during the compression test. Snapshots were taken from the video showing side and bottom views of the samples (Figure 4). During the first 2 mm compression as presented in side views at 2 mm in Figure 4, a food gel was on the artificial tongue. Further compression pushed and inserted the food gel into the artificial tongue. The insertion degree was greater for softer artificial tongues that are presented in side views at 5 mm (H15 < H7 < H0) and at 8 mm (H7 < H0) in Figure 4. If a food gel did not fracture, it was surrounded by artificial tongue when the distance became 10 mm, which was the initial thickness of the artificial tongue (Figure 4c). As the load was applied to a wide surface of the artificial tongue (“bottom” pictures of Figure 4c, the food gel was protected by the soft material from further deformation. When the upper probe was removed after the compression test, the food gel was not fractured, although some syneresis was observed as shown in the “end” picture of Figure 4c. Further, the artificial tongue recovered to its initial state (end pictures of Figure 4c). This condition is different from compression between an artificial tongue with the same-sized food gel and hard plate [16,18], in which the food gel deforms less on the wider soft material. This is the reason why the A15 gel with the lowest fracture strain exhibited a higher fracture probability (Table 3).

### 3.4. Mechanical Properties of Gellan Gum Gels Measured Using the Soft Machine

Table 5 shows the apparent moduli and some fracture properties measured with the soft machine. To calculate the apparent moluli, we assumed food gel deformation at only 2 mm compression as video observation from the side revealed that deformation of the artificial tongues was not significant. As shown in Figure 4, the bottom area was not significantly expanded at 2 mm compression; therefore, the initial sectional area (314 mm^2^) was used to calculate the apparent moluli.

The fracture forces were similar among A15, BC15, and D15, and a somewhat higher fracture force was observed in D20 than in A20 and BC20. Fracture deformation increased in the order A < BC < D for both groups. Those tendencies were similar to results in the conventional compression test (Table 2). The area ratio at fracture increased as fracture deformation increased. Further, the true stress tended to be higher in 20 series than in 15 series and decreases in the order A > BC > D.

When comparing the effect of different artificial tongues H15 and H7 for the same food gels, the fracture deformation with stiffer H15 was shorter than that with softer H7. Changes in fracture force and true fracture stress were small. Fracture work increased due to fracture deformation as the stiffness of the artificial tongue decreased. Further compression of food gel being surrounded with the artificial tongue was impossible, thus deformation mainly determined gel fracturing.

Using the softest material of H0, series of gels with original fracture force were higher than 20 N, and most BC15 and D15 with original fracture force of 16 N gels were not broken. Even for fractured gels, no clear peak at the fracture point was observed. Thus, Table 5 includes fracture data of A15 on H0 data. The fracture deformation of A15 gels that fractured at a high probability (0.83) was calculated as 10.3 mm. It suggests that the gel was inserted into the artificial tongue without fracture as shown in the side view of Figure 3. Some of D15, BC20, and D20 on H7 are suspected to have similar fractures. These cases with 10 mm or higher fracture deformation may be fractured during decompression.

## 4. Discussion

In this study, six mouthful-sized food gels (φ20 × 10 mm) were prepared with gellan gum mixtures as shown in Table 2. Previous results on gellan gels of the same size revealed that gels with a 15 N fracture force were easily fractured between the tongue and hard palate by healthy young adults [18]. When the fracture force was 20 N or higher, some gels were not broken between the tongue and palate. We adjusted the fracture force of gellan gels to about 16 N and slightly higher than 20 N. As expected, they could easily fracture and hard gels by compression between the tongue and palate. A small or large fracture strain was seen in the series A or D gels in our previous study [16], and the gels were similar to A15 and A20, or D15 and D20. As gels with the middle fracture strain were between the previous series of B and C [18], they were named BC15 and BC20. As shown in Table 2, D20 had significantly higher fracture force than A20. The other points were suitable as expected.

The maximum isometric tongue pressure of humans has been reported as in the order of 10 kPa [3,4,5,6,7,8,9,10,11,12]. We used a 50 N load cell, as it was most suitable to measure force applied on the food gels that fractured at 15–25 N (Table 2). The sectional area of food gels was first 314 mm^2^ and may expand 1.5–3.0 times during compression until fracture [19]. The artificial tongues could not fracture by the force of 50 N, the soft machine is suitable to simulate compression of soft foods between the tongue and palate.

The compression test using the soft material fractured some soft gels and did not fracture difficult type gels with tongue–palate compression. A conventional test between hard plates fracture all food gels. The soft machine was better than conventional hard machine to mimic natural oral processing of humans. The behavior of food gels between an artificial tongue and hard plate is useful for evaluation of food texture that can be mashed using the tongue. The transparent materials were stable for practical use and could demonstrate fracture or non-fracture process of food gels between the tongue and hard palate.

True fracture stress of gels having similar fracture force values tended to decrease with increasing fracture strain of original gels as deformation increased. When food gels were compressed with a soft machine, soft material deformed considerably and protected food gels from further deformation. As stiffness or apparent modulus of the artificial tongue decreased, fracture point of gels was increased. However, gel deformation in the horizontal direction was limited in the soft material. Fracture of food gels did not occur in the combination of the softest artificial tongue and food gels with the high fracture force (>20 N). Fracture force of 16 N appeared at the fracture border with the artificial tongue, suggesting an increase in the fracture of food gels with the fracture strain.

The hardest artificial tongue (H15) with a wide sectional area of 2500 mm^2^ did not deform until reaching 2 mm (20% strain) under 50 N force. The apparent modulus of the artificial tongues must be measured using small cylindrical gels using the load cell. The apparent modulus is useful parameter to compare mechanical properties of different artificial tongues, the human tongue, and food gels.

Apparent modulus of the human tongue is 12–123 kPa [16] and is in accordance with other results [3,4,5,6,7,8,9,10,11,12,13,15]. We tested three urethane gels. The hardest H15 with the apparent modulus of 161 kPa was too hard to simulate natural eating behavior using the tongue. This may be useful for an artificial gingival rather than a tongue model. As one level higher Smile Care Foods (yellow 4) are slightly harder food that can be mashed with the gums [2].

The middle H7 had an apparent modulus of 55 kPa that was just in the range of the human tongue. The apparent modulus was similar to that of a previously tested silicone rubber A5, which was 53.5 kPa [16,18]. All food gels tested were fractured with H7, as suggested in a previous report [18]. Although the artificial tongue can fracture these gels when the size is greater than the food gels, the fracture probabilities were low if the food gels and artificial tongue were of similar size [18]. It seemed too hard to mimic tongue compression, especially for the elderly individuals who require nursing care foods.

The softest H0 has an apparent modulus of 23 kPa. This value was lower than the maximum tongue pressure in healthy subjects and similar to that in frail elderly subjects [12] and in majority of elder people aged over 80 years [11]. It may be a suitable model tongue for persons with weak tongue pressure who require nursing care foods. H0 may be too soft as a tongue model of healthy adults because the human tongue tenses to mush food in the oral cavity. Because soft gels with fracture force of 15 N were on the fracture border, it is reasonable that the human tongue normally tenses to mash food in the oral cavity. A new artificial tongue that has mechanical properties closer to those of the human tongue in the tensed state is required to simulate the human behavior more precisely.

## 5. Conclusions

Soft food gels were compressed using three transparent urethane gels. The soft machine could demonstrate gel fracture between the tongue and hard palate of humans. The fracture properties obtained from the test could be useful to evaluate food texture that can be mashed using the tongue.

## Figures and Tables

**Table 1:** Formulation of gellan gels used as model soft foods.

Gellan Gel (% *w*/*w*)	A15	BC15	D15	A20	BC20	D20
Mixture of gellan gum	0.32	0.35	0.30	0.45	0.45	0.43
Low-acylated gellan gum	0.32	0.2625	0.15	0.45	0.3375	0.215
High-acylated gellan gum	0.00	0.0875	0.15	0.00	0.1125	0.215

**Table 2:** Fracture properties of gellan gels used as soft food models.

Gellan Gel	A15	BC15	D15	A20	BC20	D20
Fracture force (N)	15.7 ^a^ ± 1.1	15.9 ^a^ ± 0.6	16.8 ^a^ ± 1.2	22.5 ^b^ ± 0.9	24.2 ^bc^ ± 2.1	25.0 ^c^ ± 1.5
Fracture strain (%)	49.0 ^a^ ± 2.3	62.0 ^b^ ± 0.4	74.4 ^c^ ± 1.5	49.3 ^a^ ± 2.1	64.1 ^b^ ± 2.4	75.8 ^c^ ± 1.0

Determined at 20 °C 1 day after preparation. Mean and standard deviation values of 6 samples. Values with the same alphabetical letter within a row are not significantly different (*p* > 0.05).

**Table 3:** Characteristics of urethane gels used as artificial tongues.

Gel Sample	H0	H7	H15
Asker FP hardness	43.8 ^a^ ± 1.5	69.7 ^b^ ± 0.6	89.5 ^c^ ± 0.6
Apparent modulus (kPa)	23.3 ^a^ ± 0.3	55.1 ^b^ ± 0.9	160.9 ^c^ ± 2.2

Mean and standard deviation values of 4 or more samples. Values with different alphabetical letter within a row are significantly different (*p* < 0.05).

**Table 4:** Ratio of food gel-fracture on artificial tongue.

Gel Sample	H0	H7	H15
A15	0.83	1.00	1.00
BC15	0.33	1.00	1.00
D15	0.29	1.00	1.00
A20	0.00	1.00	1.00
BC20	0.00	1.00	1.00
D20	0.00	1.00	1.00

Probability from 3 or more samples.

**Table 5:** Mechanical characteristics of gellan gels measured by the soft machine.

Gellan Gel	A15	BC15	D15	A20	BC20	D20
on H15 artificial tongue						
Apparent modulus (kPa)	13.4 ^ab^ ± 4.7	13.2 ^abB^ ± 0.3	2.6 ^a^ ± 0.9	32.7 ^c^ ± 12.2	18.5 ^bcB^ ± 1.2	5.0 ^abA^ ± 0.8
Fracture force (N)	10.9 ^aA^ ± 3.5	12.7 ^ab^ ± 1.4	12.5 ^a^ ± 0.3	17.2 ^abc^ ± 3.5	19.3 ^bc^ ± 0.5	22.6 ^c^ ± 2.8
Fracture deformation (mm)	5.95 ^aA^ ± 0.83	6.53 ^abA^ ± 0.13	7.75 ^cdA^ ± 0.15	5.55 ^abA^ ± 0.48	6.89 ^bcA^ ± 0.19	8.17 ^dA^ ± 0.38
Fracture work (N.mm)	21.8 ^aA^ ± 3.3	27.5 ^a^ ± 1.3	23.0 ^a^ ± 1.6	32.0 ^bA^ ± 9.1	42.1 ^bcA^ ± 1.2	44.8 ^c^ ± 5.4
Area ratio at fracture to initial	1.49 ^abB^ ± 0.10	1.76 ^ab^ ± 0.10	2.00 ^ab^ ± 0.70	1.42 ^a^ ± 0.13	1.75 ^ab^ ± 0.31	2.42 ^b^ ± 0.11
True fracture stress (kPa)	27.1 ^aA^ ± 5.9	23.0 ^a^ ± 2.0	22.1 ^a^ ± 9.7	38.3 ^aA^ ± 5.3	35.9 ^a^ ± 6.3	29.6 ^a^ ± 3.3
on H7 artificial tongue						
Apparent modulus (kPa)	23.1 ^c^ ± 3.4	5.4 ^aA^ ± 4.6	2.3 ^a^ ± 1.0	20.0 ^bc^ ± 10.4	8.3 ^abA^ ± 1.5	2.6 ^aA^ ± 1.4
Fracture force (N)	13.0 ^a^ ± 1.7	12.9 ^a^ ± 4.5	14.6 ^ab^ ± 3.4	21.7 ^ab^ ± 2.1	20.8 ^ab^ ± 5.7	23.7 ^b^ ± 5.5
Fracture deformation (mm)	7.00 ^aA^ ± 0.27	7.78 ^abB^ ± 0.49	9.16 ^cB^ ± 0.85	8.82 ^bcB^ ± 0.46	9.41 ^cB^ ± 0.32	9.93 ^cB^ ± 0.37
Fracture work (N.mm)	35.5 ^abB^ ± 5.4	29.9 ^a^ ± 13.4	34.0 ^ab^ ± 10.5	71.6 ^cB^ ± 5.7	68.4 ^cB^ ± 15.7	61.6 ^bc^ ± 10.5
Area ratio at fracture to initial	1.42 ^aAB^ ± 0.04	1.60 ^ab^ ± 0.36	2.00 ^b^ ± 0.16	1.33 ^a^ ± 0.06	1.50 ^a^ ± 0.05	2.59 ^c^ ± 0.14
True fracture stress (kPa)	29.2 ^abA^ ± 3.9	25.0 ^a^ ± 5.0	23.1 ^a^ ± 4.6	52.3 ^cB^ ± 5.6	43.8 ^bc^ ± 10.8	29.3 ^ab^ ± 8.2
on H0 artificial tongue; part of A15, BC15, D15 gels fractured, A20, BC20 and D20 not fractured						
Apparent modulus (kPa)	11.3 ^a^ ± 6.9	7.0 ^aAB^ ± 2.6	2.4 ^a^ ± 1.6	10.3 ^a^ ± 3.3	9.9 ^aA^ ± 3.0	4.4 ^aA^ ± 1.1
Fracture force (N)	19.8 ^B^ ± 1.39	-	-	NF	NF	NF
Fracture deformation (mm)	10.3 ^B^ ± 0.39	-	-	NF	NF	NF
Fracture work (N.mm)	56.1 ^C^ ± 6.7	-	-	NF	NF	NF
Area ratio at fracture to initial	1.29 ^A^ ± 0.05	-	-	NF	NF	NF
True fracture stress (kPa)	49.2 ^B^ ± 4.1	-	-	NF	NF	NF

Mean ± standard deviation of at least three samples. For H0 artificial tongue, fracture properties are presented for only A15 gels with high fracture probability (0.83). Mechanical values with the same lower-case letter within a row are not significantly different among gellan samples, and those with the same upper-case letter within a column are not significantly different among artificial tongues (*p* > 0.05). Row or column without superscripts are not significantly different by one-way ANOVA (*p* > 0.05).
